# Cryptococcal Meningitis Presenting as Chronic Headache in an Apparently Immunocompetent Patient

**DOI:** 10.7759/cureus.48668

**Published:** 2023-11-11

**Authors:** Ajinkya Rahatgaonkar, Mukarram Ali, Rajat Ranka, Minakshi Dhar, Ravi Kant

**Affiliations:** 1 Internal Medicine, All India Institute of Medical Sciences, Rishikesh, IND

**Keywords:** headache with warning signs, cryptococcus neoformans, hiv/aids, chronic headache, immunocompetent, cryptococcal meningitis

## Abstract

Cryptococcal meningitis, a severe fungal infection, usually afflicts immunocompromised individuals, mainly those with acquired immunodeficiency syndrome (AIDS). However, rarely, immunocompetent individuals can develop the infection too. Here, we present a case of a human immunodeficiency virus (HIV)-seronegative individual without known immunocompromised states. This patient suffered from chronic headaches for five years before presenting to us, with multiple past consultations resulting in misdiagnoses of migraines and tension-type headaches (TTH). The patient had developed new-onset warning signs in the last month after which neuroimaging was done, which showed features of increased intracranial pressure. Cerebrospinal fluid (CSF) analysis revealed *Cryptococcus neoformans*. The patient received amphotericin B alongside flucytosine, and he underwent three therapeutic lumbar punctures (LP) to relieve symptoms from raised intracranial pressure. Within two weeks, he showed significant improvement in headaches and was discharged in a healthy state. The patient was doing fine two months post discharge. This case emphasizes the necessity of ruling out red flag signs before diagnosing primary headaches. In clinical practice, if any patient shows poor response to medications despite adequate compliance, a thorough evaluation is required to rule out serious causes of headache, with a low threshold for neuroimaging.

## Introduction

Cryptococcosis is a serious fungal disease encountered by immunocompromised patients, particularly those with acquired immunodeficiency syndrome (AIDS). An estimated 152,000 cases of cryptococcal meningitis occur among people living with human immunodeficiency virus (HIV)/AIDS worldwide each year, resulting in nearly 112,000 deaths. Most cases occur in sub-Saharan Africa [1]. Pathogenic cryptococci are basidiomycetous, encapsulated yeasts that can be subclassified into four serotypes and two species with two varieties. The serotypes are based upon capsular agglutination reactions and are designated A, B, C, or D. The genus *Cryptococcus* can be divided into two species complexes: *Cryptococcus neoformans* (serotypes A and D) and *Cryptococcus gattii* (serotypes B and C) [2]. The two varieties of *Cryptococcus neoformans* are *grubii* and *neoformans*. As per traditional knowledge, *C. neoformans* usually causes infection in immunocompromised individuals only, while *C. gattii* infection is not associated with specific immune deficits and often occurs in immunocompetent individuals. Infection is caused by the inhalation of aerosolized particles. There is a state of latency, so viable organisms are harbored for a prolonged period, possibly as granuloma. The important virulence factors in pathogenesis are polysaccharide capsules, the ability to produce melanin, and enzymes such as urease and phospholipase. The disease spectrum is composed of chronic meningoencephalitis, pneumonia, and skin or soft tissue infection. The symptoms of cryptococcal meningoencephalitis typically begin indolently over a period of one to two weeks, the most common symptoms being fever, malaise, and headache. Headache is seen in more than 75% of the patients with cryptococcal meningitis [3]. Chronic headaches and neurological symptoms should raise suspicion of cryptococcal meningitis, and the patient should be evaluated for the same. Here, we present a case of intractable headache, which was later diagnosed as chronic cryptococcal meningitis without any apparent immunocompromised state.

## Case presentation

A 52-year-old male, a laborer by occupation, active cigarette smoker with 20 pack-year smoking history and a known case of coronary artery disease, presented with an insidious-onset, intermittent, holo-cranial, poorly characterized, moderate-intensity headache persisting for the last five years. Initially, the headaches occurred one to two times a month, lasting 2-3 hours, necessitating over-the-counter analgesics for symptomatic relief. There was no associated photophobia, phonophobia, nausea, vomiting, seizures, focal neurological deficits, fever, weight loss, or visual disturbance. However, over the past month prior to presentation, he experienced acute worsening of the headache, characterized by continuous throbbing pain with increased intensity of visual analog scale (VAS) 7/10. The pain exacerbated when lying down and was alleviated upon assuming an upright position. Additionally, he reported a gradual decline in vision in both eyes, diplopia for 15 days worsening with levoversion, and 4-5 episodes of non-bilious, non-blood-stained vomiting containing food particles, without associated nausea in the four days preceding admission. These episodes were not accompanied by abdominal pain or any other gastrointestinal symptoms. The patient had no history of recurrent infections, diabetes mellitus, tuberculosis, organ transplant, malignancy, chronic liver/kidney disease, or any other known immunocompromised state. There was no history of travel in the last two years. There was no history of exposure to birds or any other pet animals.

**Figure 1:**
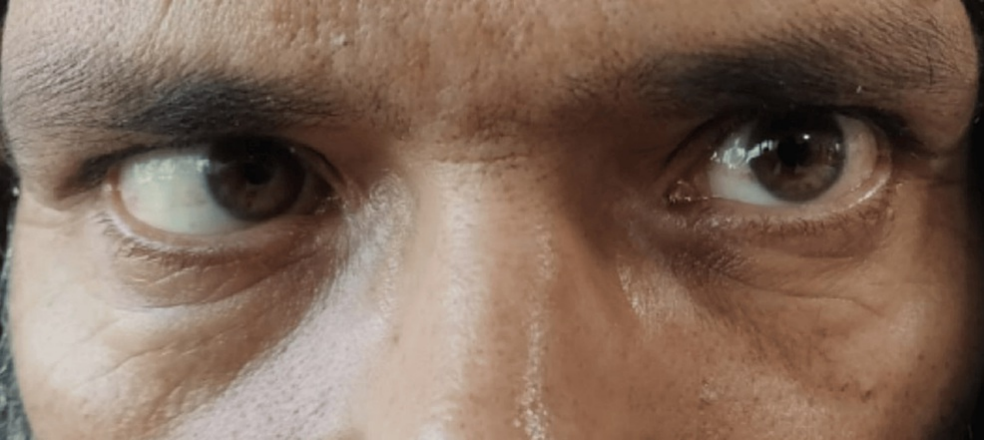
Left lateral rectus palsy: levoversion The photograph shows absent conjugate abduction of the left eye when the patient was advised to see toward the left side with normal adduction of the right eye suggestive of left lateral rectus palsy

**Figure 2:**
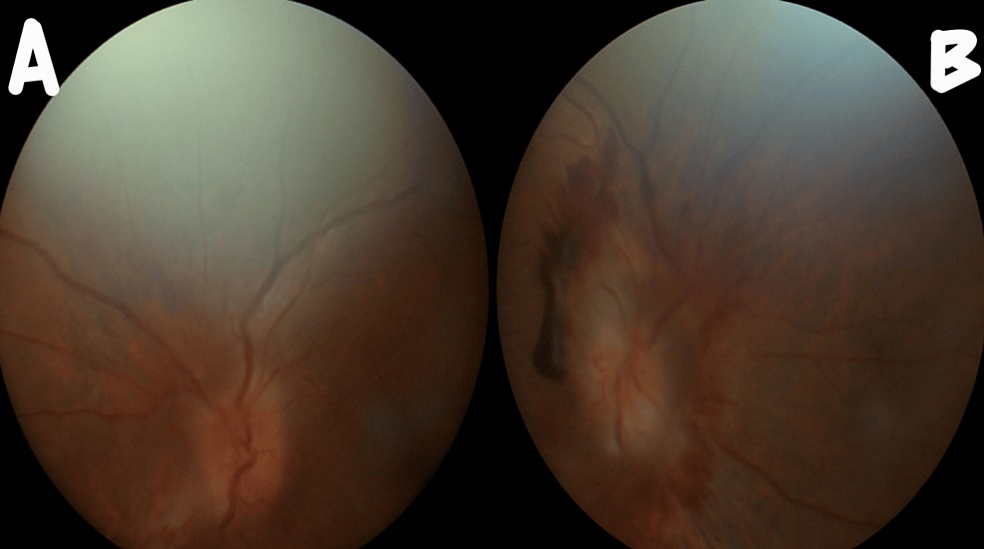
Fundus photograph: bilateral grade V papilledema The panel labelled "A" is of the right-side eye fundus image and the panel labelled "B" is of the left-side eye fundus image. There is blurring of the disc margin with circumferential halo and obscuration of all disc vessels bilaterally

Upon examination, vital parameters and sensorium were normal. The pupils were bilaterally normal in size but sluggishly reactive to light with no relative afferent pupillary defect (RAPD). Visual acuity was 6/36 in both eyes. Left lateral rectus palsy was observed (see Figure 1). Fundus examination indicated bilateral grade V papilledema (see Figure 2). Other cranial nerves and sensory-motor examination were normal. No other focal neurological deficits were identified. Deep tendon reflexes were normal, with a bilateral flexor plantar response. The patient exhibited terminal neck rigidity and a positive Kernig sign. Systemic examination did not reveal any abnormalities.

**Figure 3:**
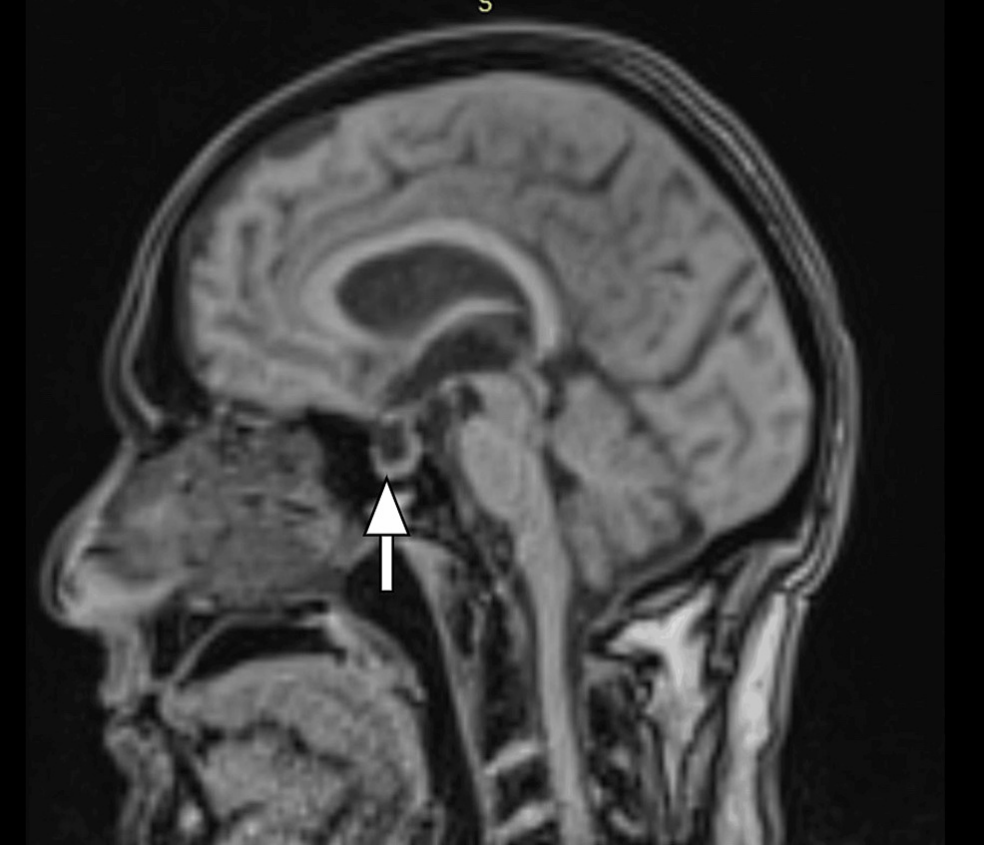
Magnetic resonance imaging (MRI) of the brain: T1 sequence, sagittal section showing partial empty sella, with normal brain parenchyma The arrow shows the ballooning of the sella

Magnetic resonance imaging (MRI) of the brain revealed a small acute lacunar infarct in the right frontal lobe. Other findings included the partial ballooning of the sella (see white arrow in Figure 3), a prominent Meckel's cave, the mild vertical tortuosity of bilateral optic nerves, a prominent subarachnoid space around bilateral optic nerves, and the flattening of the sclera at the optic cup. Overall findings were suggestive of intracranial hypertension (ICH).

A lumbar puncture (LP) was performed, and cerebrospinal fluid (CSF) analysis initially revealed mild lymphocytic pleocytosis (10/µL), elevated protein levels of 67 mg/dL, and reduced glucose levels (30 mg/dL), with corresponding capillary sugar at 74 mg/dL. The details of serial CSF analysis are shown in Table 1. CSF Gram stain, aerobic culture, and cartridge-based nucleic acid amplification test (CBNAAT) for *Mycobacterium tuberculosis* were negative. The India ink staining of CSF revealed cryptococcal-like organisms, later confirmed through culture as *Cryptococcus neoformans*. The patient was initiated on induction therapy with conventional amphotericin B (50 mg/day) and flucytosine (7.5 g/day). Serologies for hepatitis C, HIV, and hepatitis B surface antigen (HBsAg) were negative. Routine laboratory parameters were within normal limits, including absolute cluster of differentiation 4 (CD4) counts (642 cells/μL) and absolute neutrophil counts. Common immunocompromised states predisposing to cryptococcal meningitis such as sarcoidosis, chronic liver disease, diabetes, and malignancy were ruled out through appropriate laboratory and radiological investigations. No extracranial manifestations of cryptococcosis were observed. High-resolution computed tomography of the thorax did not reveal any pulmonary focus of cryptococcosis. Therapeutic LP (30-40 mL in each setting) was performed three times during the first week, and *Cryptococcus* was visualized each time on India ink and later confirmed with culture. The induction-phase therapy of 14 amphotericin doses was administered over three weeks, with intermittent breaks in therapy due to acute kidney injury. The induction phase was followed by a repeat lumbar puncture, revealing resolution on direct microscopy, as well as on culture. Clinically, the patient improved with the therapy and was discharged on high-dose oral fluconazole as consolidation-phase therapy.

**Table 1:** Serial CSF findings of the patient CSF, cerebrospinal fluid; CBNAAT, cartridge-based nucleic acid amplification test; AFB, acid-fast bacilli

Serial CSF analysis	Day 0	Day 6	Day 23
Total leucocytes (per µL)	10	Acellular	20/µL
Monomorphic cells (%)	100%	0	70%
Polymorphic cells (%)	0	0	30%
Glucose (mg/dL)	30	24	46
Protein (mg/dL)	67	130	28
India ink staining	Positive for *Cryptococcus*	Positive for *Cryptococcus*	Negative for *Cryptococcus*
Culture	C. neoformans	C. neoformans	Sterile
CSF quantity (mL)	35	30	30
Opening pressure (cm of H_2_O)	42	31	18
CSF CBNAAT for mycobacterium, negative; Gram stain, negative; and AFB stain, negative			

## Discussion

*Cryptococcus neoformans* is ubiquitous in soil contaminated with bird droppings throughout the world. Typically, it causes infection and disease in individuals experiencing some form of immunosuppression. On the other hand, *C. gattii* primarily thrives in tropical or subtropical regions, predominantly affecting immunocompetent individuals [4]. Most patients with *Cryptococcus neoformans* infection exhibit an immunocompromised state attributable to conditions such as AIDS, prolonged treatment with glucocorticoids, organ transplantation, malignancy, chronic liver disease, or rheumatological disorder sarcoidosis [5]. The infection is acquired through inhalation into the lungs. In immunocompetent individuals, it usually results in asymptomatic or mild disease. However, in immunocompromised patients, it disseminates to the central nervous system (CNS) via a hematogenous route, leading to meningitis. The most common symptoms include fever, malaise, headache, stiff neck, photophobia, and vomiting. In some cases, patients may present with severe neurological disturbances such as coma and may experience fulminant demise within days [6].

Headache is a frequent complaint prompting patients to seek healthcare. In most instances, it is attributed to either tension-type headache (TTH) or migraine. However, clinicians must be vigilant for red flag signs when present. Some points that act as warning signs and need detailed evaluation are neurological deficit; sudden-onset or abrupt headache; pattern change or recent onset of new headache; positional headache precipitated by sneezing, coughing, or exercise; and post-traumatic onset of headache.

Infections such as cryptococcal meningitis can sometimes underlie chronic headaches, as evidenced in our patient who had experienced it for five years before the diagnosis. Headache is a prevalent presentation of cryptococcal meningitis. In one Indian study, the prevalence of headaches was estimated to be as high as 75% [3].

In a multicenter study of patients with cryptococcal meningoencephalitis without HIV infection, the prevalence of various symptoms is as follows: headache, 73%; constitutional symptoms (fever, weight loss, and night sweats), 68%; and altered mental status, 42%. Although patients in this study did not have HIV infection, they did have some immunosuppression in the form of corticosteroid therapy (25%), solid organ transplant (SOT) (15%), chronic organ failure (41%), chronic lung disease (4%), hematologic malignancies (11%), and other malignancies (5%) [7]. However, there was no significant predisposing factor reported in 30% of patients with CNS involvement [8]. If we look at SOT recipients, around 8% of invasive fungal infections result from cryptococcosis. The cumulative occurrence of cryptococcosis in SOT recipients who did not utilize alemtuzumab or antithymocyte globulin was only 0.3%. In contrast, those who received a single dose of these agents experienced a 1.2% incidence of cryptococcosis, while patients who received more than one dose had a higher occurrence rate of 3.5% [9].

As such, *Cryptococcus neoformans* meningoencephalitis is rare in immunocompetent patients. The infection occurs due to high-level organism exposure or exposure to more pathogenic strains [10]. CNS disease presents with the typical signs and symptoms of chronic meningitis such as headache, fever, lethargy, sensory deficits, memory deficits, vision deficits, cranial nerve palsies, and meningismus. Patients without HIV are more likely to exhibit cryptococcoma and hydrocephalus. However, it may also manifest as chronic meningitis with a more indolent course, as seen in our patent. Indolent cases may present as subacute dementia or cause acute catastrophic vision loss. The classic characteristics of meningismus may be absent [10]. As in our case, patients may initially experience only headaches, underscoring the importance for physicians to differentiate benign causes from sinister ones. Failure to do so can lead to underdiagnosis and delayed diagnosis.

Cryptococcal disease can be diagnosed through culture, CSF microscopy for India ink, cryptococcal antigen (CrAg) detection, or CSF polymerase chain reaction (PCR). The CrAg lateral flow assay performed the best, with a CSF specificity of 99.1%. The sensitivity of culture is 94%, while it is the least for India ink staining, at 86% [11]. In clinical practice, initial diagnosis is usually made with CSF microscopy, which is later confirmed with culture, as we did for our patient.

MRI serves as an important imaging modality for assessing CNS involvement in cryptococcal infection. MRI findings encompass leptomeningeal enhancement with or without a micronodular pattern, microcystic prominence involving the temporal lobes or basal ganglia, ventriculomegaly, and brain abscess. Some patients may exhibit normal MRIs [12]. Raised intracranial pressure is a common feature in cryptococcal meningitis, with approximately 50% of patients displaying an intracranial pressure above 200 mm of water [13,14]. Intracranial hypertension (ICH) represents one of the most severe neurological complications, and morbidity and mortality rates are high with raised intracranial pressure [13,14]. The mechanism for ICH may be related to the occlusion of CSF outflow due to a high burden of yeasts and polysaccharides in the arachnoid villi. Cryptococcal meningitis causing obstructive hydrocephalus has been reported [15]. In our case, contrast-enhanced MRI of the brain exhibited an acute lacunar infarct in the right frontal lobe, a prominent Meckel's cave, a mild vertical tortuosity of bilateral optic nerves, and a prominent subarachnoid space around bilateral optic nerves, suggestive of intracranial hypertension.

The recommended initial treatment comprises liposomal amphotericin B in combination with flucytosine. Following the initial antifungal therapy, patients usually require prolonged fluconazole administration to clear the infection. Some individuals will necessitate lumbar punctures to alleviate increased pressure in the brain [16]. In our case, we also performed serial lumbar punctures; the opening pressure gradually decreased and was within the normal range in the last session, coinciding with symptomatic improvement.

The disease is uniformly fatal without treatment. Even with treatment, mortality is estimated to be 20%, mostly due to elevated intracranial pressure [16]. The differential diagnosis of cryptococcosis should be considered in patients presenting with headaches and neurological symptoms, prompting early imaging and CSF analysis for timely diagnosis and treatment.

## Conclusions

Cryptococcal meningitis due to *C. neoformans* is a relatively rare condition among apparently immunocompetent individuals. When frank immunosuppression is not evident, less-considered sources of minor immunosuppression should be explored. These include, but are not limited to, chronic corticosteroid use, alcoholism, diabetes mellitus, chronic liver or kidney disease, and sarcoidosis. This case suggests that for cryptococcal meningitis to occur, the immunosuppression or typical features of meningitis are not necessary. It can present with a chronic headache, so any headache with warning signs should always be evaluated appropriately with a low threshold for neuroimaging.
